# Al_2_O_3_/TiO_2_ inverse opals from electrosprayed self-assembled templates

**DOI:** 10.3762/bjnano.9.23

**Published:** 2018-01-19

**Authors:** Arnau Coll, Sandra Bermejo, David Hernández, Luís Castañer

**Affiliations:** 1 Research Group in Micro and Nano Technologies, Electronic Engineering Department, Universitat Politècnica de Catalunya, Jordi Girona 1-3, 08034 Barcelona, Spain

**Keywords:** electrospray, metamaterials, photonic crystals

## Abstract

The fabrication of high optical quality inverse opals is challenging, requiring large size, three-dimensional ordered layers of high dielectric constant ratio. In this article, alumina/TiO_2_–air inverse opals with a 98.2% reflectivity peak at 798 nm having an area of 2 cm^2^ and a thickness of 17 µm are achieved using a sacrificial self-assembled structure of large thickness, which was produced with minimum fabrication errors by means of an electrospray technique. Using alumina as the first supporting layer enables the deposition of TiO_2_ at a higher temperature, therefore providing better optical quality.

## Introduction

Periodic structures comprised of different refractive index materials having a lattice constant matching the wavelength of the incident light are known as photonic crystals (PCs). PCs exhibit diffraction properties in one, two and three dimensions with applications in several optoelectronic devices such as dichroic mirrors, optical switches, lasers, biosensors or strain sensors [1–5]. The performance of these devices is mainly related to the lattice periodicity, the quality and the size of the ordered structure and the refractive index contrast.

The fabrication of PCs with dimensions of hundreds of micrometers and larger with sufficient thickness that are free of defects is challenging and not easy to achieve with the conventional fabrication techniques [6–7]. Although self-assembly is one of the preferred deposition techniques (compared to alternative top-down approaches based on lithography or holography), the present state of the current techniques [8–13] either leads to various randomly oriented domains [14] or to limited defect-free areas, and furthermore, they are typically very slow fabrication processes. In addition, a review of recent literature shows that opals made of polystyrene or poly(methyl methacrylate) (PMMA) [15–24] nanoparticles can be orderly assembled in larger areas and thicknesses, however, such materials do not achieve the same optical performance as inverse opals, due to issues with structure definition [25].

Inverse opals can be created using colloidal crystals (CCs) as templates to build close packed assemblies of air spheres. Several materials have been used as templates, for example, metal oxides [26–29], semiconductors [30] or silicon [31–34]. Polymeric nanoparticles can also be used as templates, as they can be deposited in an ordered way and can be either dissolved or burned after the main structural material has been deposited. The technological procedure, however, suffers from limitations of the temperature compatibility of the structural material deposition process with the maximum temperature that the polymeric nanoparticles can sustain, which is typically below 90–100 °C. This low temperature reduces the choices of materials that have suitable optical properties for a given application [35–36]. As an example, TiO_2_ deposited at <150 °C has a lower refractive index than TiO_2_ deposited at >200 °C.

In previous works [37–40] we have shown how an electrospray process of nanofluids can be used to deposit in a very short time very well ordered and with few defects layers (colloidal crystals) of polystyrene (of good quality and size) or silicon dioxide nanoparticles with dimensions typically several hundreds of micrometers with a close packed, face-centered cubic, three-dimensional order. In parallel we have shown the use of Al_2_O_3_ as a good candidate for the inverse opal supporting layer regarding the low temperature deposition capacity and mechanical properties [41–42]. The main purpose of this article is to apply this technique to the fabrication of inverse opals using the 3D polystyrene nanoparticle template which enables the creation of large scale, large thickness, self-assembled structures with minimum fabrication errors and Al_2_O_3_/TiO_2_ as a structural layer infiltrated through the voids. This is a two-step atomic layer deposition (ALD) process in which the polymeric template is eliminated after the deposition of the alumina layer and before the ALD deposition of the titania layer.

## Results and Discussion

**Figure 1:**
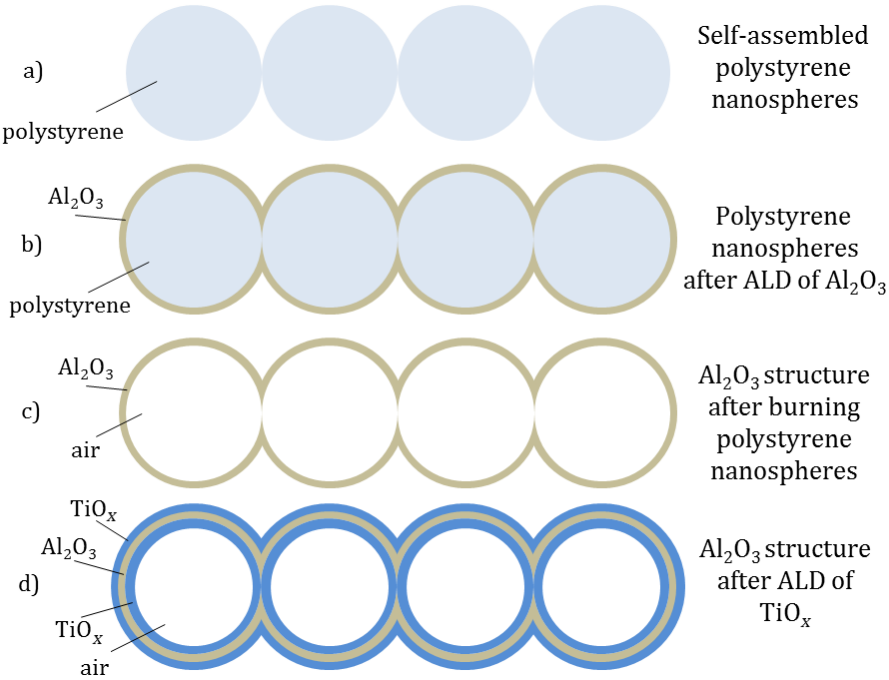
Schematic side view of the fabrication process.

The fabrication process of the Al_2_O_3_/TiO_2_ inverse opals is schematically outlined in Figure 1 where the starting step can be seen in Figure 1a and consists of the electrospray deposition of the template layer of ordered polystyrene nanoparticles. Only a side view of one row of four nanoparticles is shown for clarity. The electrospray process of a nanofluid containing polystyrene nanospheres is described in the Experimental section below.

The second step, shown in Figure 1b, consists of the deposition of a thin, conformal layer of Al_2_O_3_ in an ALD reactor at 80 °C. Such a low deposition temperature preserves the integrity of the polystyrene nanoparticle template.

The third step, shown in Figure 1c is the selective elimination of the template by raising the temperature in an oven up to 450 °C, which is high enough to burn the polystyrene particles. As it can be seen, at this stage of the process, the device is a 3D periodic structure of alumina and spherical voids. The shape of the voids depends on the initial order of the polystyrene nanoparticle layer.

At this point the sample is already an inverse opal having a refractive index contrast of 1.7/1 between alumina and air. The next step of the process is the deposition of a conformal ALD layer of TiO_2_. Titania conformally covers the alumina layer as shown in Figure 1d. At this point, the structure is an inverse opal of a composite Al_2_O_3_/TiO_2_ layer with air voids.

**Figure 2:**
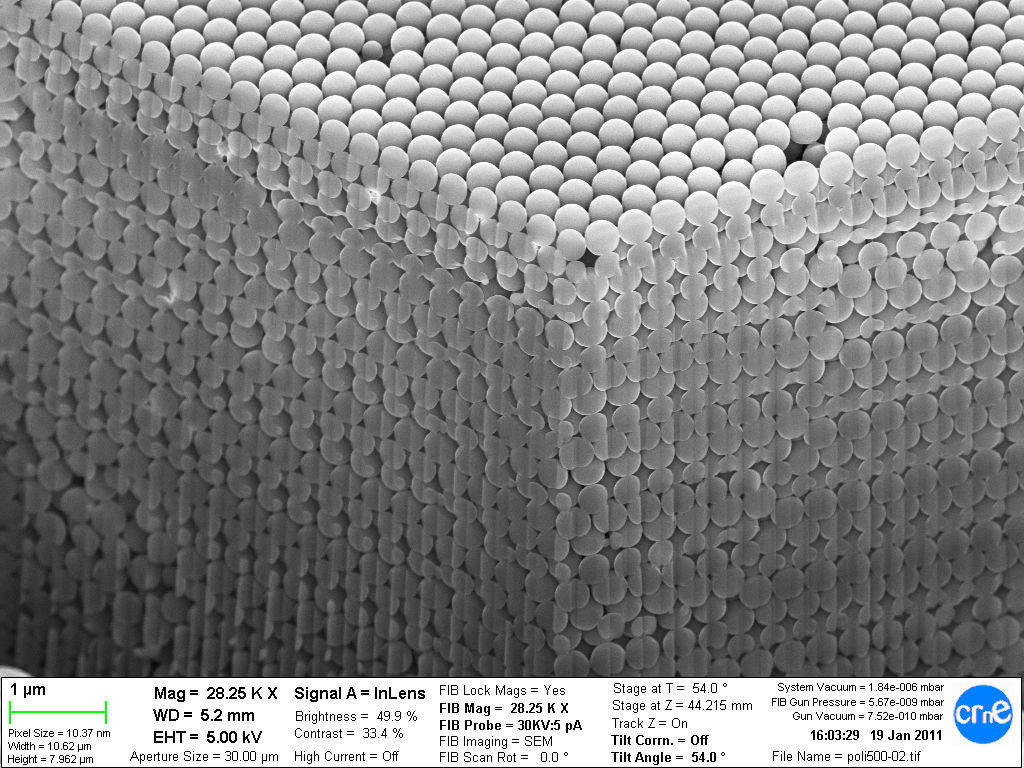
Two cross sections at 90° of a 360 nm polystyrene nanoparticle colloidal crystal. Reprinted with permission from [37], copyright Coll et al.

The result of the first fabrication step is shown in Figure 2 where up to 50 layers of 360 nm polystyrene nanoparticles can be seen. The layer is almost free of defects besides some missing beads that can be identified in the top surface. A focused ion beam (FIB) drill in two orthogonal vertical planes confirms that the order is fully three-dimensional. The total thickness of the obtained structures was 17 µm. The total area of the sample was 2 cm^2^.

### Electrospray technique

The electrospray technique, described in more detail in [43] has two main phases. In the first phase, the solution is pumped through the needle at a controlled rate using an infusion pump. The needle is positively biased at a high voltage typically between 2 and 10 kV (depending on the needle-to-substrate distance) and the substrate is biased to a voltage between −500 to −1000 V using a high voltage bipolar power source. The high electric field created causes the fluid at the tip of the needle to adopt a cone shape, a so-called Taylor cone [44]. At the tip of the Taylor cone, a small jet is formed that breaks into fine droplets. During this first phase, the droplets travel towards the substrate and land on top of it. At this point, some liquid is left on the substrate even after the infusion pump is switched off. In our experiments, the power supply providing the bias to the needle and substrate as well as the nitrogen flow are still switched on. This means that while the remaining liquid dries, the electric field is still on. This electric-field-assisted drying process is believed to be fundamental for the orderly self-assembly of the nanoparticles due to the combination of several physical mechanisms – the most relevant for the self-assembly being the dielectrophoretic force [45–46].

In our experiments, the setup was adjusted using an off-the-shelf polystyrene solution in water (Corpuscular, Inc.), applying +9000 V to the needle and −1000 V to the substrate with a separation of 14 cm and a pumping rate of 2.2 mL/h. The mentioned conditions produced a lattice of nanoparticles exhibiting a good ordering in random hexagonal close packed (HCP) domains. These domains are typically 100 µm wide with dislocations in between, and no cracks were present.

### Atomic layer deposition

The second step of our fabrication procedure uses the polystyrene structure shown in Figure 2 as a template in which other suitable materials can be deposited. In our case, alumina was first deposited as it is a material that can be easily conformally deposited in thin layers of a few nanometers thick and has the advantage that the deposition temperature is compatible with the maximum temperature that the polystyrene nanoparticles can withstand (<100 °C).

The ALD process uses trimethylaluminum (TMA) as the precursor gas. The temperature of the reactor is 80 °C, which avoids any possible damage to the polystyrene structures. The whole process produces a 20 nm conformal alumina layer. At this point of the process, the sample is an ordered layer of 360 nm polystyrene nanoparticles covered by a 20 nm thin layer of Al_2_O_3_.

**Figure 3:**
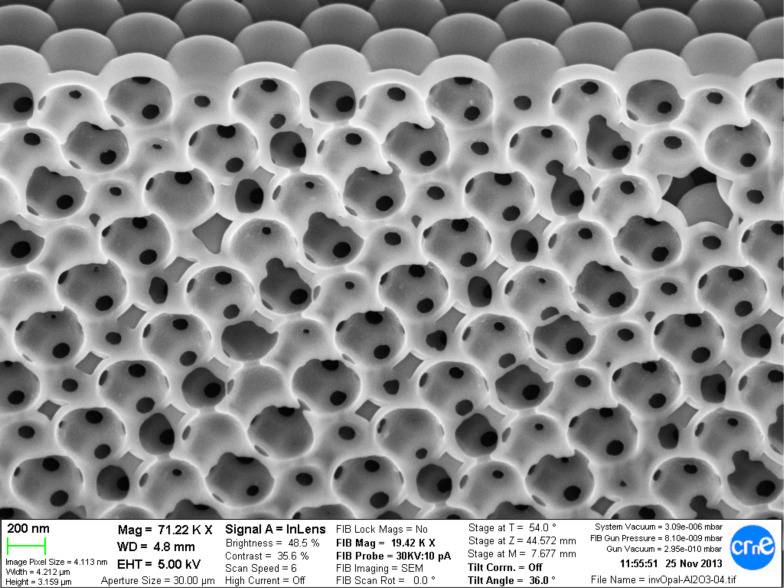
Cross section of Al_2_O_3_ shells after complete removal of polystyrene nanoparticles. Conformal shells which were formed in the gaps between nanoparticles are seen. The dark contrast areas in the shells are locations where the former polystyrene structure contact points result in uncovered areas.

In the third step, the polystyrene nanoparticles are burnt in a furnace by applying a temperature ramp from room temperature to 450 °C at 5 °C/min. The sample is kept at 450 °C for two hours. After the polystyrene beads are burnt away, the resulting inverse opal is formed inside the cavities as a layer between the nanoparticles . At the same time, inside the shells there are distinct areas where there is no Al_2_O_3_, which correspond to the previous contact points between alumina nanoparticles as shown in Figure 3.

**Figure 4:**
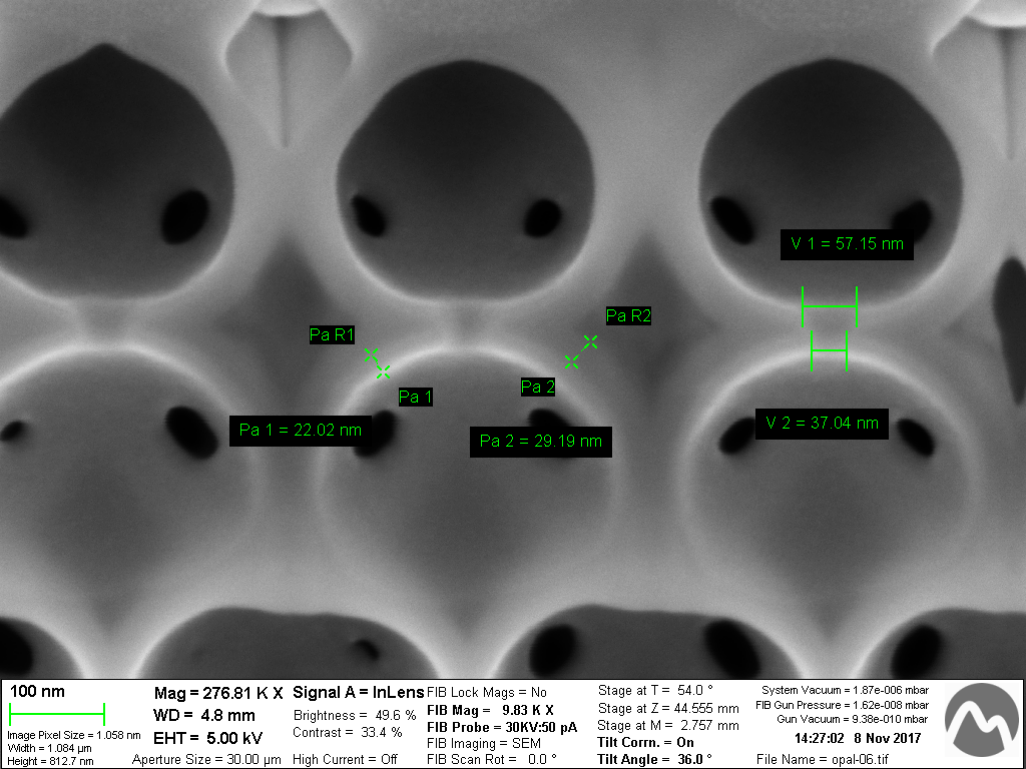
Cross section of Al_2_O_3_/TiO_2_ shells after TiO_2_ layer deposition. Conformal shells are observed covering the Al_2_O_3_ layer. Pa1 stands for the thickness of TiO_2_ and Pa2 stands for the thickness of Al_2_O_3_.

The fourth step is the conformal deposition of an ALD TiO_2_ layer around the alumina structure. This is performed using titanium osopropoxide (TIPT) as the precursor gas. The temperature of the precursor is 80 °C and the reactor temperature is 200 °C. A 20 nm conformal layer of TiO_2_ is produced covering the alumina structure as shown in Figure 4. The resulting structure is very similar as the one shown in Figure 3 but with a much higher refractive index contrast, improving the optical response. The covering of TiO_2_ is restricted to inside the particles because the ALD gases penetrate the structure through the connecting holes.

After checking the TiO_2_ deposition results (Figure 4), the conformity of both layers could be confirmed, and at the same time, the adhesion of the ALD deposition in two steps was clearly confirmed. The deposition of TiO_2_ at 200 °C improved the characteristics of the layer, as can also be seen [35–36].

### Optical response

**Figure 5:**
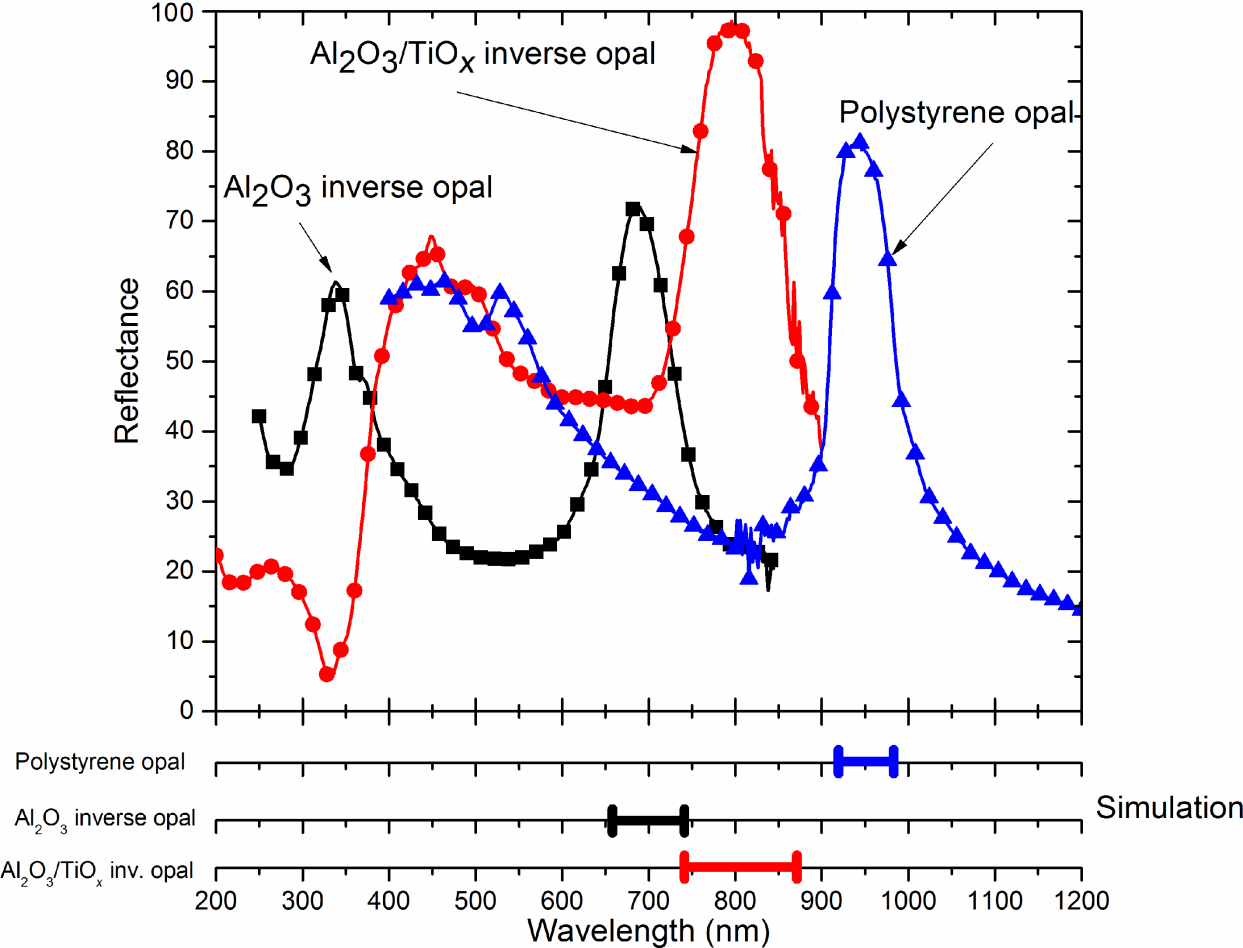
Reflectance measurements showing that the high reflection peaks closely match with the expected peaks calculated by simulation (in the lower part of the figure).

To determine the optical quality of the resulting inverse opal, the reflectance of the fabricated samples has been measured over an area of 36 mm^2^ using a Fourier transform infrared (FTIR) spectrometer with an integrating sphere. We have conducted characterization measurements on the three steps of our process: (A) after step 1 in Figure 1a, (B) after step 3 in Figure 1c and (C) after step 4 in Figure 1d. The reflectance values as a function of the wavelength are shown in Figure 5.

Looking to the plot corresponding to the measurement for step (A) related to the “polystyrene opal”, a broad peak of 81% reflectance located around 940 nm can be seen. The value of this center wavelength of the reflection peak is consistent with theoretical calculations based on the plane wave expansion method using MPB simulation software [47] that predicts a relative stop band in the normal direction to the surface of the crystal for an ordered random HCP lattice. This is consistent with our measurements, and similar results are found elsewhere [37]. A secondary gap is also found centered at 470 nm wavelength.

The measurements performed for step (B) that corresponds to the Al_2_O_3_ inverse opal are shown in Figure 5. A 72% reflectance peak centered at 686 nm and a secondary peak close to 340 nm are observed. The blueshift of the peaks is due to the different 3D structure of the inverse opal compared to the polystyrene opal and the material itself which has been substituted from polystyrene to alumina. Finally, measurements performed for step (C) correspond to the Al_2_O_3_/TiO_2_ inverse opal. As it can be seen, the main reflectivity peak is centered at 798 nm and exhibits a very high reflectance value of 98.2%. The secondary peak shows 67.8% reflectance and is centered at 448 nm. This structure was also simulated using the MPB software assuming that the voids between the nanoparticles were covered by Al_2_O_3_ and TiO_2_ at a 50/50 vol %. The simulation returned a value of the center wavelength of the first peak around 805 nm, which is very close to the experimental one.

It is relevant to mention that the reflectivity results of our measurements are given in absolute units using barium sulphate as a reference and not in relative values to the maximum response of the sample, as is the case in most published results (see for example [35–36]). Moreover, our results have been obtained using an illumination spot size of 36 mm^2^ with an integration sphere instead of using a guided light with smaller spot size focusing on the best point over the whole PC with the aim to achieve the best results, as is the case in [33].

## Conclusion

In conclusion, this paper shows how an electrospray technique can significantly contribute to advance the mass production fabrication of large area (>2 cm^2^), large thickness (>17 µm) inverse opals with very good reflectivity properties in the NIR spectral region using a double layer Al_2_O_3_/TiO_2_ colloidal crystal created by self-assembled polystyrene nanoparticles as a template. The results achieved were accomplished using low temperature processing, producing a large sample without the presence of cracks and a high absolute value of the reflectance measured with a large spot size, indicating the high optical quality of the final structure.

## Experimental

The setup for the electrospray process is based in an infusion pump and an OMNIFIX 5 mL syringe, both from B. Braun SA (Melsungen, Germany), a Hamilton needle (600 μm outer and 130 μm inner diameter; Hamilton, Bonaduz, GR, Switzerland), a high-voltage bipolar power source of −15 kV to +15 kV (Ultravolt, Ronkonkoma, NY, USA), and finally, an off-the-shelf nanofluid with 360 nm polystyrene nanoparticles at 25 g/mL concentration. The entire process was performed inside a glove box in order to control the drying atmosphere using nitrogen flow.

For the ALD process used to deposit Al_2_O_3_, a system from Cambridge Nanotech Savannah was used and TMA (Sigma-Aldrich) was used as a precursor gas. For TiO_2_ deposition, TIPT (Sigma-Aldrich) was used as the precursor gas.

For the structural characterization, scanning electron microscopy (SEM) was employed and an SEM-FIB (NEON 40, Carl Zeiss) was used for the creation of trenches used to inspect inside the samples.

In order to assess the quality of the process, the samples were measured using a spectrophotometer from Shimadzu (UV3600 UV–vis–NIR, Kyoto, Japan) and an ISR-3100 integrating sphere attachment of 3 × 12 mm beam area.
